# The relationship between internet addiction and daytime sleepiness in nursing students: a moderated mediation model

**DOI:** 10.3389/fpsyt.2026.1756841

**Published:** 2026-04-22

**Authors:** Ying Li, Dan Bu, Keyan Yuan, Piao Xia

**Affiliations:** 1 College of Sports Science, Jishou University, Jishou, Hunan, China; 2 Department of Neurology, The Third Affiliated Hospital of Naval Medical University, Shanghai, China; 3 Operating Room, Nanjing Gaochun People’s Hospital, Nanjing, Jiangsu, China

**Keywords:** daytime sleepiness, depression, internet addiction, nursing students, sleep quality

## Abstract

**Background:**

Internet addiction is highly correlated with daytime sleepiness, but the underlying mechanisms between the variables need to be further explored. The aim of this study was to investigate sleep quality as a mediating factor and depression as a moderating factor to further elucidate the potential risk factors between internet addiction and daytime sleepiness in nursing students.

**Method:**

A self-report survey was conducted among 1,578 nursing students from eight universities in China. The survey included measures of internet addiction, sleep quality, daytime sleepiness, and depression. Descriptive and correlation analysis of these variables was performed, and a moderated mediation model was constructed.

**Results:**

Internet addiction was positively correlated with sleepiness, sleep quality, and depression between nursing days. Sleep quality played a partial mediating role in the relationship between internet addiction and daytime sleepiness in nursing students, and depression strengthened the relationship between internet addiction and sleep quality pathway in a moderated mediation model.

**Conclusion:**

This study further revealed the psychological mechanism of the relationship between internet addiction and sleepiness in nursing. Sleep quality was a mediating factor for this relationship, and depression may enhance the strength of the relationship between internet addiction and sleep quality variables.

## Introduction

Sleep is essential for adolescents’ physical growth, emotional stability, and cognitive function (1). However, daytime sleepiness, defined as uncontrolled drowsiness impairing daily vigilance (2), is highly prevalent. Incidence rates range from 12% to 16% in the general population (2), exceed 40% in children and adolescents (3, 4), and reach 24%–39% in college students (5). Daytime sleepiness contributes to inattention, academic decline, negative emotions, and reduced life satisfaction (6–8), potentially escalating to self-injury and suicide. Nursing students face heightened risks due to increasing clinical practice schedules across academic years and night shifts that sacrifice sleep for daytime practical classes and nighttime theoretical education (9). Consequently, they are particularly susceptible to daytime sleepiness.

Internet addiction, characterized by excessive uncontrollable use disrupting daily function (10), is recognized by the *Diagnostic and Statistical Manual of Mental Disorders, 5th Edition* (*DSM-5*) as a potential mental health problem affecting academic, social, and occupational functioning (11), with broader consequences for physical health, relationships, and productivity (10, 12). College students are particularly susceptible due to their psychological and physiological characteristics (13). Internet addiction profoundly impacts their mental health, academic performance, relationships, and quality of life, leading to impaired cognition, attention deficits, reduced learning efficiency, and increased health risks (14–19). Critically, internet addiction is associated with sleep problems and deprivation (20). Internet use increases daytime sleepiness and careless behavior, causing time management issues, irregular meals, and impaired daily living and school performance (21, 22). Notably, Nowak et al. (23) and Singh et al. (24) confirmed the direct relationship between internet addiction and daytime sleepiness.

Sleep quality, defined by sleep onset, maintenance, and restoration, is a vital indicator of sleep status (25). It facilitates academic achievement, as students with better sleep demonstrate superior coping strategies, adaptation, and performance (26, 27). Conversely, internet addiction, often stemming from unmet social needs as a psychological compensation, correlates with declined sleep quality (28). Excessive internet use and addiction increase the likelihood of poor sleep by 2.4 and 4.39 times, respectively (29), a significant correlation confirmed by meta-analysis (30).

Individual traits, particularly depression, can strengthen variable relationships and exacerbate negative outcomes (31). Nursing students face career pressures and heavier clinical burdens, resulting in higher depression incidence than other majors (32, 33). High depression involves lacking social support, negative coping, and heightened stress perception, leading to sleep problems (34, 35). Additionally, internet addiction correlates with depression, stress, and anxiety (36, 37), with severe addiction predicting depressive mood (38, 39). Consequently, depression may reinforce the relationship between internet addiction and sleep quality in nursing students, worsening adverse psychological and behavioral outcomes.

**Figure 1:**
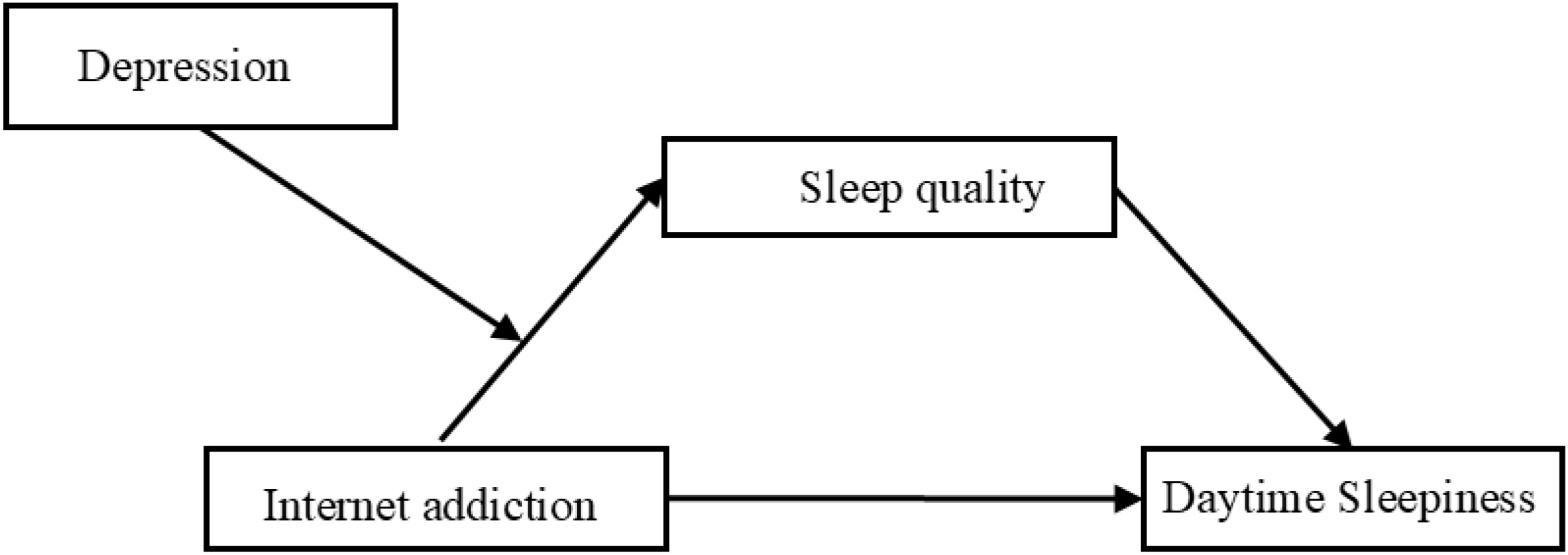
Hypothesized moderated and mediation model.

In summary, while previous studies have robustly demonstrated the association and predictive relationship between internet addiction and daytime sleepiness, research focusing on nursing students remains relatively scarce. To further advance this field and explore underlying psychological mechanisms, we aimed in this study to examine the mediating role of sleep quality in the relationship between internet addiction and daytime sleepiness among nursing students, as well as the moderating role of depression. Accordingly, a hypothetical path model was constructed for this study (see **Figure 1**).

## Methods

### Participants

A cross-sectional survey was conducted among nursing students from eight universities in Shandong, Jiangxi, Shanghai, and Liaoning using convenience sampling from August to September 2024. Before distribution, the researchers explained the purpose of the study to all participants, informing them of the main content and confidentiality of the survey data. The electronic questionnaire was distributed through nursing teachers, and a statement of informed consent was attached to the questionnaire’s cover. Participants will only be able to proceed with the survey after consent, while those who refuse will be directed to the opt-out page. Informed consent was obtained from all participants. The study was done anonymously and voluntarily within 20 min. We calculated the sample size as per the following formula:


$$n=\frac{\mu^{2\alpha }{/}_{2}\pi (1-\pi )}{\delta^{2}}$$

where α = 0.05, μα/2 = 1.96, and δ = 0.05. In order to ensure sufficient sample size, π = 43% was set for calculation (33, 40). Considering 20% non-response rates, we found that the minimum sample size was 476. Based on the *post-hoc* power analysis, the statistical power was estimated at 99%, indicating a 99% probability of correctly detecting a true effect when one exists (41). A total of 1,651 students completed the survey. After excluding respondents who were too short in response time or whose response patterns were too short, valid data were obtained for 1,578 participants. The questionnaire response rate was 95.578%.

### Measures

#### Center for epidemiological studies depression scale

The flow-call Depression Scale was developed in 1977 and is widely used to screen depressive symptoms (42). There are 20 items on the scale, graded 0–3, with a total score of 0–60 points. The assessment is conducted according to the actual situation in the latest week. No more than 1 day, 1–2 days, 3–4 days, and 5–7 days correspond to “no or almost no,” “rarely,” “often,” and “almost always,” respectively. The scale includes four dimensions: depressive mood, positive mood, physical disorder, and interpersonal relationship. The evaluation criteria of this scale are as follows: a total score ≤15 indicates no depressive symptoms, a score of 16–19 indicates possible depressive symptoms, and a score ≥20 indicates specific depressive symptoms (42). In this study, the Cronbach’s α for the sample was 0.963.

#### Pittsburgh sleep quality index scale

This scale was developed by Buysse et al. (43). to evaluate the subjects’ sleep quality in the last 1 month. In this study, the Pittsburgh Sleep Quality Index (PSQI) scale revised by Liu et al. (44) was adopted, including 18 items and seven dimensions; each dimension was 0–3 points, and the total score was 0–21 points. The higher the score, the worse the sleep quality. The Cronbach’s α coefficient of this scale was 0.817.

#### Internet addiction

The Internet Addiction Scale was compiled by K.S. Yong of the University of Pittsburgh (45). It has been widely used to measure internet addiction (46). The scale consists of 20 items, each with five choices (almost none to always), assigned a score of 1–5. It includes four dimensions: network damage, compulsive symptoms, network relationship addiction, and the influence of destructive emotions. The subjects’ internet use was determined according to the scale’s total score. The higher the score, the more serious the degree of addiction to the internet. The score of 20–49 was everyday internet use, and the score of 50–100 was internet addiction. In this study, the Cronbach’s α for the sample was 0.941.

#### Epworth sleepiness scale

The Epworth Sleepiness Scale (ESS) translated and revised by Peng et al. (2011) was adopted to evaluate the daytime sleepiness of college students (47). The scale consisted of eight items and was scored with four points, 0 indicating “never dozing off” and 3 indicating “often dozing off”. The total score ranged from 0 to 24 points. The higher the score, the more serious the degree of daytime sleepiness. Previous studies have shown that this scale has good reliability and validity in college students (48). In this study, the Cronbach’s α coefficient of this scale was 0.858.

### Statistical analysis

All analyses were performed using IBM SPSS Statistics Version 26.0. The mean and standard deviation of continuous variables were used to calculate internet addiction, depression, self-control, and sleep quality scores. Chi-square test was used to compare internet addiction with different demographic characteristics. In order to provide the basis for the selection of appropriate mediating variables, we carried out partial correlation analysis to determine the correlation between variables. PROCESS macros implemented in SPSS software (IBM Corporation, Armonk, NY, USA) (49) were used to analyze the mediation model. The PROCESS macro plugin uses 5,000 iterations of self-lifting new samples to evaluate model tests and 95% confidence interval estimates. If the 95% CI does not include 0, the relationship is significant. *p*< 0.05 was considered statistically significant.

## Results

### Common method biases test

The standard method biases test was used to control for the problem of common method bias; this study used Harman’s one-way test for common method bias (50). The results showed that the first common factor explained only 27.273% (<40%) of the variance. This indicates no significant standard method bias in this study despite using the questionnaire.

### Participants’ characteristics

**Table 1** presents information on demographic characteristics and the results of univariate analysis. Among them, 331 (20.976%) were men and 1,247 (79.024%) were women. Of the 1,578 participants, 18.694% (*n* = 295) met the criteria for daytime sleepiness. The single-factor analysis of this study showed that drinking was the main reason affecting daytime sleepiness (*p*< 0.05).

**Table 1:** Sociodemographic characteristics of participants.

Variables	N	No	Yes	χχ ^2^/t	p
Age				0.703	0.704
17–19	720	588	132		
20–22	776	626	150		
>23	82	69	13		
Gender				1.275	0.259
Female	1,247	1,021	226		
Male	331	262	69		
Educational level				0.763	0.382
Associate	710	584	126		
Bachelor’s	868	699	169		
Drink alcohol				6.880	0.009*
Yes	1,396	1,148	248		
No	182	135	47		

*P<0.05.

### Preliminary correlation analyses

The correlation between depression, internet addiction, daytime sleepiness, and sleep quality is shown in **Table 2**. Daytime sleepiness was positively correlated with internet addiction, sleep quality, and depression (*r* = 0.130, *p*< 0.001; *r* = 0.496, *p*< 0.001; *r* = 0.404, *p*< 0.001). Internet addiction was positively correlated with sleep quality and depression (*r* = 0.163, *p*< 0.001; *r* = 0.117, *p*< 0.001). In addition, sleep quality was positively correlated with depression (*r* = 0.446, *p*< 0.001).

**Table 2:** Correlational analyses (*n* = 1,578).

Variable	Daytime sleepiness	Internet addiction	Sleep quality	Depression	Gender	Age	Educational level
Depression daytime	–						
Internet addiction	0.130**						
Sleep quality	0.496**	0.163**					
Depression	0.404**	0.117**	0.446**				
Gender	0.032	0.004	0.028	0.010			
Age	0.026	0.022	−0.014	0.031	0.042		
Educational level	0.031	−0.010	−0.023	−0.037	0.047	0.330**	
Drink alcohol	0.133**	0.056	0.136**	0.027	0.311**	0.029	0.043

**p<0.001.

### Mediation analysis

The results are shown in **Table 3**. After controlling for gender, age and other factors, internet addiction significantly predicted daytime sleepiness (β = 0.043, SE = 0.009, *p*< 0.001). When sleep quality was included as a mediator, internet addiction still significantly predicted daytime sleepiness among nursing students (β = 0.017, SE = 0.008, *p*< 0.05). As shown in **Table 4**, internet addiction among nursing students was directly associated with daytime sleepiness (β = 0.017, 95% CI: 0.002 to 0.032), and sleep quality played a significant mediating role in this relationship (β = 0.026, 95% CI: 0.017 to 0.036).

**Table 3:** Mediation model test.

Variables	Daytime sleepiness			Sleep quality			Daytime sleepiness		
	β	SE	*t*	β	SE	*t*	β	SE	*t*
Gender	−0.103	0.286	−0.361	−0.070	0.152	−0.461	−0.040	0.252	−0.160
Age	0.097	0.20	0.488	−0.054	0.106	−0.507	0.146	0.176	0.830
Educational level	0.210	0.236	0.891	−0.105	0.125	−0.836	0.304	0.207	1.468
Drink alcohol	1.779	0.365	4.869	0.989	0.194	5.095**	0.889	0.324	2.747*
Internet addiction	0.043	0.009	4.953**	0.029	0.005	6.282**	0.017	0.008	2.193*
Sleep quality							0.899	0.042	21.569**
*R* ^2^	0.034			0.044			0.254		
*F*	10.955**			14.338**			89.359**		

*p<0.05, **p<0.001.

**Table 4:** Total, direct, and indirect effects.

Paths	Effect	Boot SE	Bootstrap 95% (CI)		Relative mediation effect
			Boot LL CI	Boot UL CI	
Total effect of X on Y	0.043	0.009	0.026	0.060	
Direct effect of X on Y	0.017	0.008	0.002	0.032	39.53%
Indirect effects (X→M→Y)	0.026	0.005	0.017	0.036	60.47%

LLCI, the lower limit of *B* in 95% confidential interval; ULCI, the upper limit of *B* in 95% confidential interval.

### Moderated and mediation analysis

**Figure 2:**
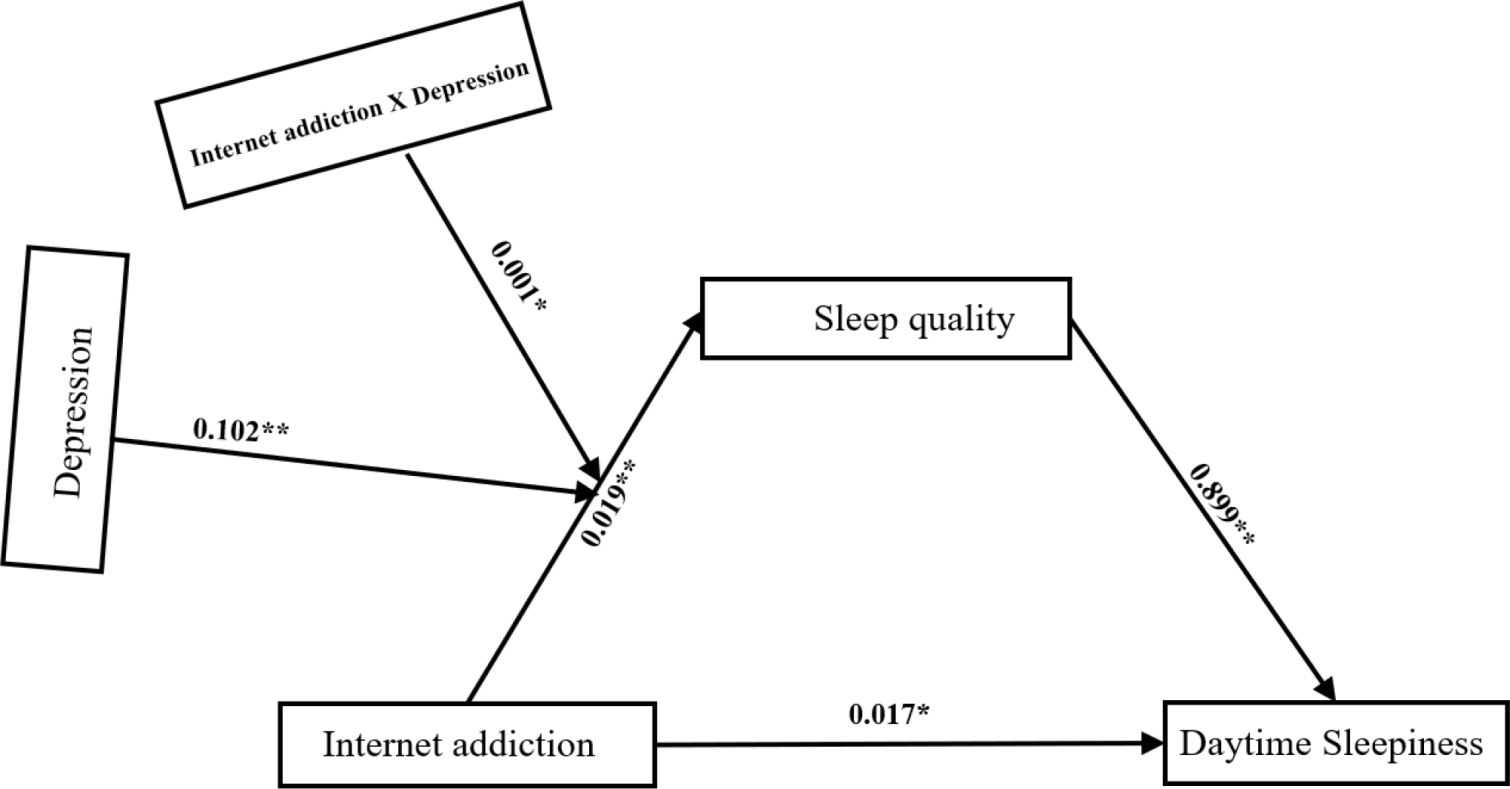
Moderated and mediation model. *P<0.05, **P<0.001.

**Figure 3:**
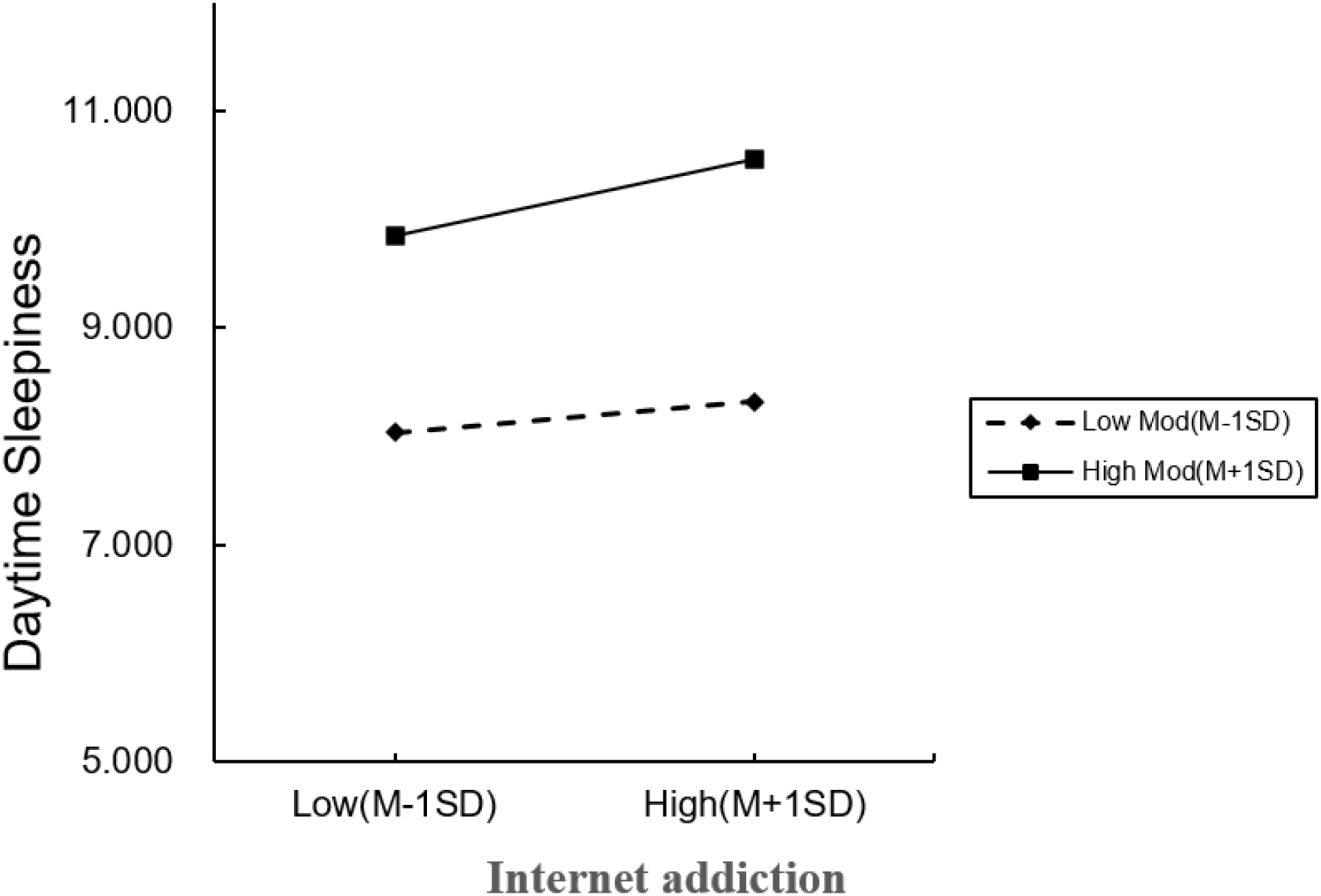
Simple slope plot.

After controlling for covariates, mediated model analysis showed that the prediction effect of all pathways in the mediated model remained significant (internet addiction predicted sleep quality: β = 0.019, *p*< 0.001; depression was predicted by sleep quality: β = 0.102, *p*< 0.001). In addition, internet addiction significantly predicted daytime sleepiness (β = 0.017, *p*< 0.05). Sleep quality significantly predicted daytime sleepiness (β = 0.899, *p*< 0.001). See **Table 5** and **Figure 2**. The simple slope plot further illustrates that internet addiction exerts a stronger influence on daytime sleepiness at higher levels of depression. See **Figure 3**.

**Table 5:** Moderated and mediation model.

Variables	Sleep quality			Daytime sleepiness		
	β	SE	*t*	β	SE	*t*
Gender	−0.083	0.137	−0.606	−0.040	0.252	−0.160
Age	−0.138	0.096	−1.442	0.146	0.176	0.830
Educational level	0.004	0.113	0.035	0.304	0.207	1.468
Drink alcohol	0.924	0.175	5.292**	0.889	0.324	2.747*
Internet addiction	0.019	0.004	4.368**	0.017	0.008	2.193*
Depression	0.102	0.006	18.423**			
Internet addiction × Depression	0.001	0.004	2.299*			
Sleep quality				0.899	0.042	21.569**
*R* ^2^	0.229					
*F*	66.675**					

*p<0.05, **p<0.001.

## Discussion

This study examined the relationship between internet addiction, depression, sleep quality, and daytime sleepiness in nursing students. The findings revealed a positive correlation between internet addiction, depressed sleep quality, and daytime sleepiness, all of which were statistically significant. After controlling for demographic variables, we found that sleep quality mediated the relationship between internet addiction and daytime sleepiness among nursing students. At the same time, depression moderated internet addiction and sleep quality, confirming our initial hypothesis.

Our study found that the incidence of sleepiness between nursing days was 18.694%, which was slightly higher than the Demir et al. (51) study. Consideration may have a particular relationship with the difference in the major of the survey students. Daytime sleepiness can lead to a decline in nursing function, academic performance, and other physiological and psychological problems (2). For nurses in practice, inter-day sleepiness will affect the working status of nursing students and increase clinical work errors and adverse events (52). In addition, alcohol consumption in our study is the cause of sleepiness between nursing days, which is consistent with previous research findings (53). Studies have found that 30% of college students will use alcohol to help them sleep, and alcohol will also inhibit physical functions, resulting in excessive daytime sleep symptoms (54). The results of this study suggest that reducing drinking behavior can help nursing students manage daytime sleepiness.

Our study confirmed the positive correlation between internet addiction and sleepiness between nursing days, which is consistent with previous research results (55). In the study, Demir et al. (51) found that students with daytime sleepiness had shorter sleep times and higher scores of internet addiction, and students with daytime sleepiness had a higher proportion of delayed night sleep due to internet use. Kapahi et al. (56) determined that people who use the internet have sleep problems until midnight, and college students often stay up late because of internet use. Nursing students are highly dependent on the use of social networks or mobile phones, especially before going to bed. They will worry that they will not receive information as soon as they fall asleep, causing anxiety and anxiety (57), which will delay the time to fall asleep. They will not be able to put down their mobile phones, which will reduce the sleep-inducing effect of their bed and bedroom. As a result, the nerves are excited (58), and the time to fall asleep will be extended. Reduced sleep duration leads to daytime sleepiness.

Our study supports the hypothesis that sleep quality mediates the relationship between internet addiction and daytime sleepiness in nursing students. Previous research has found a strong association between internet addiction and sleep quality (30), and the relationship between sleep quality and daytime sleepiness is also well-supported (59).

According to the sleep displacement theory, individuals with internet addiction spend considerable time online, particularly at night, which significantly encroaches upon sleep time and compromises sleep quality (60). Empirical studies confirm that college students with internet addiction are more likely to delay bedtime and report poorer sleep quality (61). Additionally, blue light emitted by electronic screens can disrupt physiological rhythms; long-term exposure inhibits melatonin secretion and delays circadian rhythms, thereby affecting sleep quality (62, 63). Sleep quality is also a significant predictor of daytime sleepiness (64): the poorer an individual’s sleep quality, the higher their level of daytime sleepiness (65). According to the dual-process theory of sleep regulation, sleep results from the interaction between the homeostatic and circadian systems, and their coordinated function ensures good sleep quality and stable sleep rhythms (66). However, internet addiction can cause sleep delay and circadian rhythm disruption, leading to sleep problems such as decreased sleep quality and impaired daytime functioning (67). Nighttime sleep structure disturbances—including sleep deprivation, reduced deep sleep, and repeated awakenings—can reduce sleep quality and contribute to daytime sleepiness (68). The primary cause of daytime sleepiness is the fragmentation of sleep structure resulting from repeated micro-awakenings during nocturnal sleep (68).

As previously hypothesized, depression may strengthen the relationship between internet addiction and sleep quality. Nursing work itself is unique; the gender of nursing professionals is relatively single, the process of specialization is complicated and slow, and undergraduate nursing students in the study or practice of professional cognitive education are slightly inadequate, which will inevitably produce depression and other negative emotions. Studies have found that addiction symptoms have the effect of self-medication (69), and adolescents are in a period full of pressure and challenges. When they face depression, they are prone to distract their attention and relieve their depression through internet addiction (70). To get rid of negative emotions and escape the learning pressure, in reality, students with depression become addicted to the internet world. Some studies have found that after forming an internet addiction, social difficulties and sleep problems are likely to occur, creating a vicious circle (71). If repeated without intervention, individuals will aggravate internet addiction, and the emergence of internet addiction will lead to sleep quality problems. Therefore, high levels of depression strengthened the relationship between internet addiction and sleep quality, as expected in this study.

### Recommendations

It is recommended that nursing educators identify at-risk nursing students and provide psychosocial support when necessary. Specifically, for students with elevated levels of depression, early psychological screening and personalized intervention are crucial. Educators should establish routine mental health monitoring mechanisms to promptly identify high-risk signals. Furthermore, it is recommended that training on healthy internet use, sleep promotion, and depression management be provided to nursing students on an ongoing basis (72). Nursing institutions can incorporate relevant content into compulsory curricula or continuing education programs. Through various formats such as workshops, specialized lectures, and online modules, these programs can help students establish healthy lifestyles and stress-coping strategies. Additionally, considering the unique stressors and shift schedules during clinical internships, administrators should optimize scheduling to prevent the accumulation of excessive fatigue. Ultimately, improving the sleep and psychological status of nursing students will not only enhance their academic performance and physical and mental health but also reduce the risk of fatigue-induced medical errors in future clinical practice, thereby ensuring nursing service quality and patient safety.

### Limitations

Several limitations of this study should be acknowledged. First, the cross-sectional design necessitates caution when interpreting the results. Although this approach identifies associations at a specific time point, it precludes definitive causal inferences, thereby limiting the ability to discern causal dynamics. Second, the study relies on self-reported data, which may introduce potential biases, particularly recall bias. The accuracy and authenticity of participants’ responses could be influenced by their current emotional state, memory capacity, and personal perceptions, potentially affecting the reliability and validity of the collected data.

## Conclusion

This study examined the interrelationships among internet addiction, sleep quality, and daytime sleepiness in nursing students. The results demonstrated that sleep quality mediated the relationship between internet addiction and daytime sleepiness, whereas depression moderated the association between internet addiction and sleep quality. Consequently, nursing educators and administrators should prioritize mitigating the adverse effects of daytime sleepiness, particularly among students exhibiting elevated levels of depression.

## Data Availability

The original contributions presented in the study are included in the article/supplementary material. Further inquiries can be directed to the corresponding author.
